# Validation of diagnostic screening test for pharmacogenomic targets for thiopurine drugs in indian pediatric acute lymphoblastic leukemia patients

**DOI:** 10.3389/fphar.2025.1714797

**Published:** 2025-12-08

**Authors:** Gratial Theres Joseph, Sandeep Kumar Swain, Tejas Somwanshi, Aarishi Singh, Manpreet Kaur, Raj Kamal, Himanshu Dhanda, Prashant Kumar, Upasana Kaushik, Baseer Noor, Saurabh Sharma, Pranay Tanwar, Sufian Zaheer, Sumita Chaudhry, Prashant Prabhakar, Amitabh Singh, Bhavika Rishi, Aroonima Misra

**Affiliations:** 1 ICMR-NICHDR (National Institute of Child Health and Development Research), New Delhi, India; 2 Manipal University of Higher Education (MAHE), Manipal, India; 3 Hamdard Institute of Medical Sciences and Research (HIMSR), New Delhi, India; 4 Indian Council of Medical Research, New Delhi, India; 5 Laboratory Oncology Unit, Dr. BRA-IRCH, AIIMS New Delhi, India; 6 Department of Pathology, Vardhman Mahavir Medical College and Safdarjung Hospital, New Delhi, India; 7 Department of Hematology, Vardhman Mahavir Medical College and Safdarjung Hospital, New Delhi, India; 8 Department of Pediatrics, Vardhman Mahavir Medical College and Safdarjung Hospital, New Delhi, India

**Keywords:** acute lymphoblastic leukemia (ALL), thiopurine toxicity, NUDT15 c.415C>T, TPMT*3C, Pharmacogenetics, ARMS-PCR, 6-mercaptopurine, genotype-guided therapy

## Abstract

**Background:**

Thiopurines such as 6-mercaptopurine (6-MP) are central to maintenance therapy for pediatric acute lymphoblastic leukemia (ALL), yet their narrow therapeutic index frequently causes dose-limiting myelosuppression in genetically susceptible patients. Variants in *NUDT15* and *TPMT* are major pharmacogenetic determinants of thiopurine intolerance, particularly in Asian populations. Despite CPIC recommendations for pre-emptive genotyping, routine pharmacogenetic testing is seldom implemented in India due to high costs and limited access to sequencing-based platforms.

**Aim:**

To develop, standardize, and validate a rapid, low-cost tetra-primer ARMS-PCR assay for simultaneous detection of the most clinically relevant variants—*NUDT15* c.415C>T and *TPMT 3C* (c.719A>G)—prioritized through comprehensive *in silico* analysis.

**Methods:**

Missense SNPs in *NUDT15* and *TPMT* were screened using SIFT, PolyPhen-2, PROVEAN, Meta-SNP, and SNPs&GO to identify high-impact variants. A multiplex ARMS-PCR assay was optimized and applied to 61 pediatric ALL samples. Genotyping results were validated by Sanger sequencing. Clinical correlations included hematologic toxicity, 6-MP dose intensity, and blast-percentage dynamics.

**Results:**

In-silico prioritization consistently identified *NUDT15* c.415C>T and *TPMT 3C* as the most deleterious and clinically actionable variants. Among 60 successfully genotyped patients, *NUDT15* variants were detected in 16.7% (9 C/T and 1 T/T), while *TPMT 3C* heterozygosity was observed in 3.3%; no double-mutants were identified. ARMS-PCR showed 98.4% overall diagnostic accuracy relative to Sanger sequencing (sensitivity 90.9%, specificity 100%). *NUDT15*-variant carriers exhibited significantly reduced 6-MP dose intensity (median 0.50 vs. 0.79; p < 0.0001). Blast-percentage analysis demonstrated marked reduction from baseline to follow-up (median 75%–15%; p = 0.0001), consistent with expected treatment response.

**Conclusion:**

The validated ARMS-PCR assay provides a reliable, rapid, and cost-effective platform for simultaneous *NUDT15* and *TPMT 3C* genotyping, demonstrating strong concordance with sequencing and clear clinical relevance. Its affordability and minimal infrastructure requirements make it suitable for integration into routine pre-treatment workflows in India, enabling genotype-guided thiopurine dosing and reducing the risk of treatment-related toxicity. This assay supports a scalable path toward equitable implementation of pharmacogenomics in resource-limited pediatric oncology settings.

## 1 Introduction

Acute lymphoblastic leukaemia is the most common pediatric malignancy, and 6-mercaptopurine remains a cornerstone in its maintenance therapy. But its narrow therapeutic index brings out considerable hematologic toxicity, which includes leukopenia, neutropenia, myelosuppression especially among genetically susceptible individuals (Relling et al., 2013; Lee et al., 2021).

In large measure, these toxicities have been found to result from inherited differences of two enzymes involved in the metabolism of the thiopurines, namely, thiopurine S-methyltransferase (TPMT) and nudix hydrolase 15 (NUDT15) (Maillard et al., 2024).

Thiopurines are a group of purine analogs commonly used in the treatment of haematological malignancies, autoimmune diseases, and inflammatory bowel diseases. As prodrugs, thiopurines are metabolized to thioguanine nucleotides that are then incorporated into DNA, becoming cytotoxic and thus immunosuppressive (Ho et al., 2017). Recent studies have also pointed out the role of oxidative stress and reactive oxygen species generation as additional factors contributing to thiopurine-induced cytotoxicity (Verma et al., 2023).

Two inactivation pathways act together to limit excessive accumulation of TGN and consequent marrow suppression. TPMT catalyzes S-methylation of thiopurine metabolites, while NUDT15 hydrolyzes thioguanosine triphosphate (TGTP) to its monophosphate derivative, preventing misincorporation into DNA (Maillard et al., 2024; Valerie et al., 2016). Loss-of-function variants in either gene markedly reduce enzymatic activity, resulting in elevated intracellular TGTP concentrations and enhanced drug toxicity (Khera et al., 2019; Tanaka and Saito, 2021). Both enzymes participate in different yet complementary detoxification routes; thus, simultaneous assessment of the TPMT and NUDT15 variants offers a more comprehensive evaluation of thiopurine tolerance.

There is marked variation in the prevalence and clinical relevance of TPMT and NUDT15 variants across populations. In Caucasian and African populations, TPMT polymorphisms, in particular 3A and 3C, are the major determinants of thiopurine metabolism, and genotype-directed dosing has become routine (Relling et al., 2013; Lee et al., 2021). However, these are much rarer in East and South Asian populations, among whom the NUDT15 c.415C>T (p.Arg139Cys) variant has emerged as a major risk factor for thiopurine intolerance (Maillard et al., 2024; Valerie et al., 2016).

In Indian paediatric ALL cohorts, NUDT15 c.415C>T has been reported in approximately 9%–10.7% of cases, while TPMT*3C appears in 3%–3.5% (Pratt et al., 2022; Relling et al., 2019). These frequencies resemble those of East Asian populations in which NUDT15 variants (10%–12%) better predict myelotoxicity than TPMT mutations (Maillard et al., 2024; Khera et al., 2019).

According to the joint CPIC consensus, tier-1 pharmacogenetic variants that warrant clinical testing include *2, *3A, 3B, 3C, and 4 for TPMT, and 2, 3, and 6 for NUDT15. While data on the complete spectrum of variants in Indian populations is scant, current evidence suggests that the most clinically actionable polymorphisms are NUDT153 and TPMT3C (Pratt et al., 2022; Relling et al., 2019).

Clinical Carriers of NUDT15 or TPMT variants exhibit significantly reduced tolerance to thiopurines. Inadequate TPMT activity results in excessive accumulation of the active TGN, leading to dose-dependent marrow suppression and a risk of infection, whereas NUDT15 variants are associated with severe early-onset leukopenia, gastrointestinal toxicity, and alopecia even at moderate doses. In a North Indian cohort, all leukopenias were confined to carriers of the NUDT15 variant (p < 0.0001), and nearly two-thirds developed clinically significant toxicity during therapy (Aşlar Öner 2024). In an Indian inflammatory bowel disease cohort, carriers of NUDT15 c.415C>T faced a nearly 19-fold higher risk of leukopenia, while TPMT variants were rare (Shah et al., 2017).

The complexity of molecular regulation in precision oncology, illustrated by such signaling molecules like the NONO protein in breast cancer (Lone et al., 2023), is an indication that pharmacogenetic testing should be included in therapeutic protocols. Genotype-directed dosing may minimize adverse reactions and improve treatment outcomes in paediatric ALL.

Despite CPIC recommendations to perform TPMT and NUDT15 genotyping and reduce thiopurine doses to approximately 10 mg/m^2^/day for poor metabolisers, this is not a routine practice in India. Most genetic studies are confined to the detection of classical driver mutations, and the unavailability of affordable genotyping facilities has limited access to pharmacogenetic-based treatment. The development of a fast and inexpensive PCR-based screening could thus help in improving the safety and feasibility of treatment at resource-poor centers.

The present study integrates clinical observations and *in silico* analyses to identify NUDT15 c.415C>T and TPMT*3C as functionally important variants in Indian pediatric ALL. We report the development and validation of a rapid, low-cost ARMS-PCR assay for simultaneous genotyping and correlate identified variants with hematologic toxicity, dose modification, and therapy interruption. These observations support the incorporation of TPMT and NUDT15 screening into pre-treatment evaluation protocols and enable safer, genotype-guided thiopurine therapy in the Indian population.

Although multiple deleterious SNPs were identified *in silico*, the NUDT15 c.415C>T (p.Arg139Cys) and TPMT*3C (c.719A>G) variants were prioritized based on their consistently high pathogenicity scores across five computational tools, their known clinical association with thiopurine intolerance in Asian populations, and their prevalence in Indian cohorts. These two variants account for the majority of thiopurine dose modification cases in Indian pediatric ALL, justifying their selection for assay development.

## 2 Methodology

### 2.1 Retrieval and identification of canonical missense SNPs

For maximum coverage and higher sensitivity in diagnostic screening, a comprehensive *in silico* analysis was performed to identify deleterious single nucleotide polymorphisms (SNPs) within the NUDT15 and TPMT genes. SNP datasets were retrieved from the NCBI dbSNP database (https://www.ncbi.nlm.nih.gov/snp/), yielding extensive entries for both genes. After deduplication, a subset of missense mutations was shortlisted for pathogenicity assessment. Multiple computational prediction tools were employed to evaluate the potential functional impact of non-synonymous coding SNPs (nsSNPs), including Sorting Intolerant From Tolerant (SIFT), PolyPhen-2, PROVEAN, PANTHER, SNPs&GO, and Meta-SNP. These tools were used to assess evolutionary conservation, structural and functional impacts, and disease association, thereby prioritizing variants with the highest likelihood of clinical relevance for subsequent genotyping.

We aim for predictive accuracy to ensure reliable identification of functionally relevant variants and to minimize false-positive or false-negative interpretations. Accurate predictions enhance the clinical relevance of genotyping by guiding safe and effective therapy adjustments. To strengthen predictive accuracy, we employed additional bioinformatic tools for cross-validation, including PolyPhen-2 (http://genetics.bwh.harvard.edu/pph2/), PROVEAN (http://provean.jcvi.org/), PANTHER (http://www.pantherdb.org/), SNPs&GO (http://snps.biofold.org/snps-and-go/snps-and-go.html), and Meta-SNP (http://snps.biofold.org/meta-snp/). These tools were used to assess the potential structural, functional, and disease-associated impacts of the shortlisted variants, integrating evolutionary conservation, protein domain information, and predictive algorithms to support variant prioritization for clinical genotyping.

### 2.2 Sample collection

For assay standardization and validation, we conducted a pilot study comprising 61 peripheral blood samples from pediatric patients diagnosed with acute lymphoblastic leukemia (ALL) at Safdarjung Hospital between 2023 and 2025Diagnosis was confirmed using standard hematological and molecular criteria. Samples were obtained under aseptic conditions and handled according to institutional protocols, with ethical approval and compliance with the Declaration of Helsinki, ensuring patient confidentiality and sample integrity for downstream analyses.

### 2.3 Standardization assay

Genomic DNA was extracted from 4 mL of EDTA-anticoagulated peripheral blood using the QIAamp DNA Blood Mini Kit (Qiagen, Germany), and quantified using a NanoDrop Spectrophotometer (Thermo Scientific, United States).

#### 2.3.1 Primer designing and genotyping by ARMS-PCR

**FIGURE 1:**
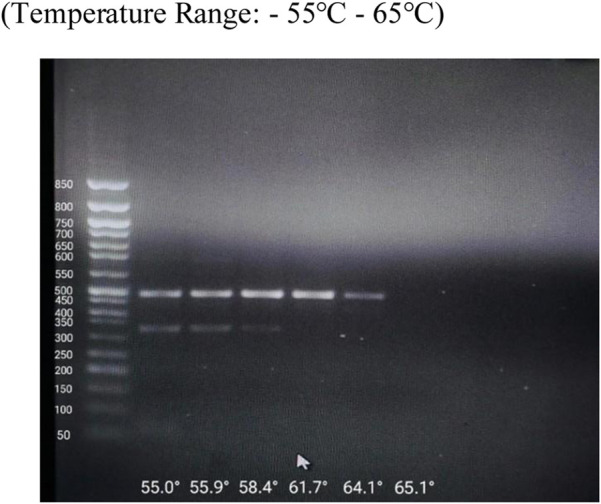
Optimization of Annealing Temperature by Gradient PCR.

Tetra-primer ARMS-PCR primers for NUDT15 and TPMT SNPs were designed using the PRIMER1 web tool. Reference sequences were obtained from NCBI dbSNP, and SNP positions were validated from published data. Primer design parameters were optimized to ensure allele-specific amplification, including length, melting temperature, GC content, and amplicon size (Supplementary Table S2). Each set comprised two outer primers for a common control fragment and two inner allele-specific primers generating distinct amplicon sizes, enabling clear differentiation of homozygous and heterozygous genotypes via gel electrophoresis (Figure 1). Expected amplicon sizes were 233 bp (common), 117 bp (wild-type), and 155 bp (mutant). Lyophilized primers (Eurofins Genomics, India) were reconstituted with nuclease-free water; stock solutions were stored at −20 °C, and working solutions were prepared by 10-fold dilution.

To detect the presence of *NUDT15* c.415C>T and *TPMT* c.719A>G variants, Amplification Refractory Mutation System-PCR (ARMS-PCR) was employed using a tetra-primer design. Each 25 µL reaction mixture consisted of 10 ng genomic DNA, 12.5 µL of AmpliTaq Gold 360 Master Mix (Applied Biosystems, Foster City, CA, United States), and 3 µL of gene-specific primer mix, with nuclease-free water added to reach the final volume. Thermal cycling was performed on a Veriti 96-Well Thermal Cycler (Prima-duo® Thermal Cycler, HiMedia, India)) with initial denaturation at 95 °C for 10 min, followed by 35 cycles of denaturation (95 °C for 1 min), annealing (62 °C for *NUDT15*, 57 °C for *TPMT*) for 45 s, and extension (72 °C for 45 s), ending with a final elongation at 72 °C for 10 min.

A total of 61 pediatric ALL patient samples were included for genotyping analysis. To validate assay performance, positive controls for NUDT15 and TPMT variants were generously provided by Dr Prateek Bhatia lab,from PGI Chandigarh. Negative controls comprised DNA samples from healthy individuals with no prior history of hematological disorders. Family members of variant-positive patients were also screened to evaluate possible hereditary transmission. PCR products were resolved on 2% agarose gel using 1× TAE buffer at 100 V for 70 min. Gels were stained with ethidium bromide, and band patterns were visualized using ultraviolet transillumination. The lower limit of detection (LOD) was assessed using serial dilutions of template DNA (10 ng–0.1 ng).

### 2.4 Validation using patient samples and clinicopathological correlation

Patient-derived DNA samples were analyzed alongside positive controls (obtained from PGI Chandigarh) and negative controls (DNA from healthy individuals) to validate the ARMS-PCR assay. Specificity was confirmed by excising representative PCR amplicons from agarose gels and subjecting them to Sanger sequencing on an automated genetic analyzer at Biologia Research India. Sequence alignment and variant confirmation were carried out using SnapGene 2.3 software.

Genotypic data were integrated with patient clinical profiles for clinicopathological correlation, including hematological toxicity, treatment interruptions, and therapeutic response parameters. Statistical analysis was performed using Kaplan–Meier survival analysis for overall survival estimation, with comparisons assessed via log-rank test. Chi-square test was applied to evaluate associations between genotypes and clinical characteristics, Mann–Whitney test to compare 6-MP dose intensity between NUDT15-positive and negative groups, and t-test to assess differences in blast percentage at baseline and follow-up. A p-value <0.05 was considered statistically significant.

Genotyping by ARMS-PCR and Sanger sequencing was conducted independently by two separate researchers blinded to each other’s results. The ARMS-PCR analysis was performed at ICMR-NICHDR, while sequencing confirmation was outsourced to Biologia Research India. Final results were compared only after completion of both analyses to ensure unbiased assessment.

We selected NUDT15 c.415C>T and TPMT3C for assay development because these two variants are the most prevalent and clinically actionable determinants of thiopurine intolerance in Indian pediatric ALL patients. Both are classified as CPIC Tier-1 variants, have strong and well-established associations with early-onset myelosuppression, and represent the variants most likely to benefit routine pre-treatment screening in the Indian population. TPMT2 and TPMT*3B were not included, as these variants are rare in India and were not part of our *in silico* prioritization or validation pipeline (Pratt et al., 2022).

### 2.5 Blast cell percentage analysis

Bone marrow aspirates from ALL patients were collected at baseline and post-induction follow-up. Smears were Wright–Gimesa stained, and blast cells were quantified as a percentage of nucleated cells by two independent hematopathologists. Differences between baseline and follow-up were analyzed using the Mann–Whitney U test with 95% confidence intervals.

### 2.6 NUDT15 and TPMT genotyping and 6-MP dose intensity

DNA was extracted from EDTA blood, and NUDT15 c.415C>T and TPMT*3C genotyping was performed using ARMS-PCR. Patients were grouped as wild-type or mutant. 6-MP dose intensity was calculated as actual/planned dose (60 mg/m^2^/day). Median dose intensities were compared between groups using the Mann–Whitney U test (p < 0.05 considered significant).

## 3 Results

**FIGURE 2:**
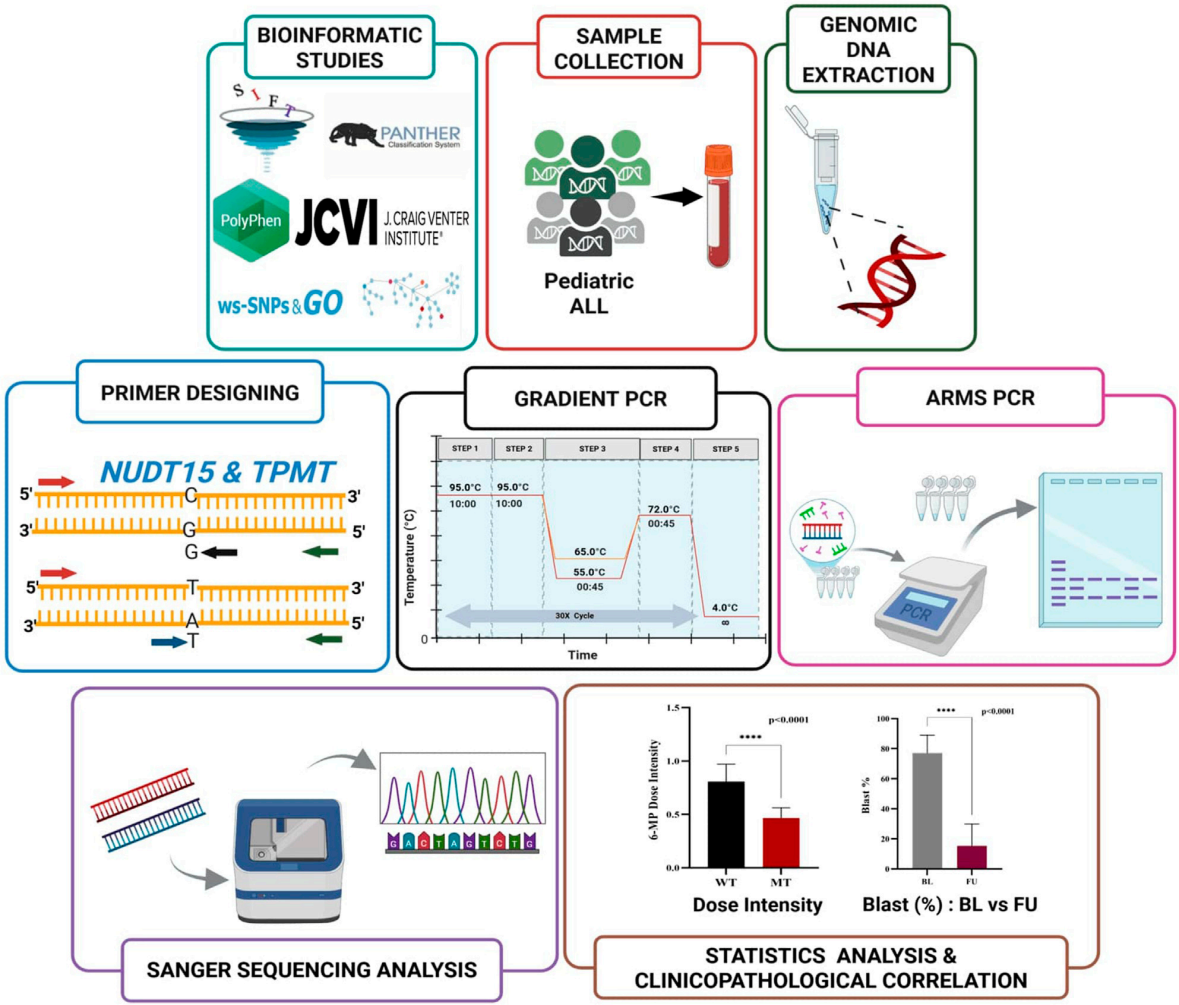
Schematic workflow of ARMS-PCR assay for NUDT15 and TPMT genotyping.

Even though epidemiological studies revealed high prevalence of NUDT15 c.415C>T and TPMT*3C in Indian Paediatric ALL cohorts, we conducted a comprehensive *in silico* screening analysis to validate their predicted functional impact, systematically evaluated all other potentially relevant variants in these genes and confirm that these epidemiologically common variants are indeed the most functionally deleterious among all possible SNPs. This dual approach ensured and robust our assay targets variants with both high clinical prevalence and confirmed pathogenic potential (Figure 2).

### 3.1 In silico characterization of NUDT15 and TPMT variants data retrieval and SNP selection

**FIGURE 3:**
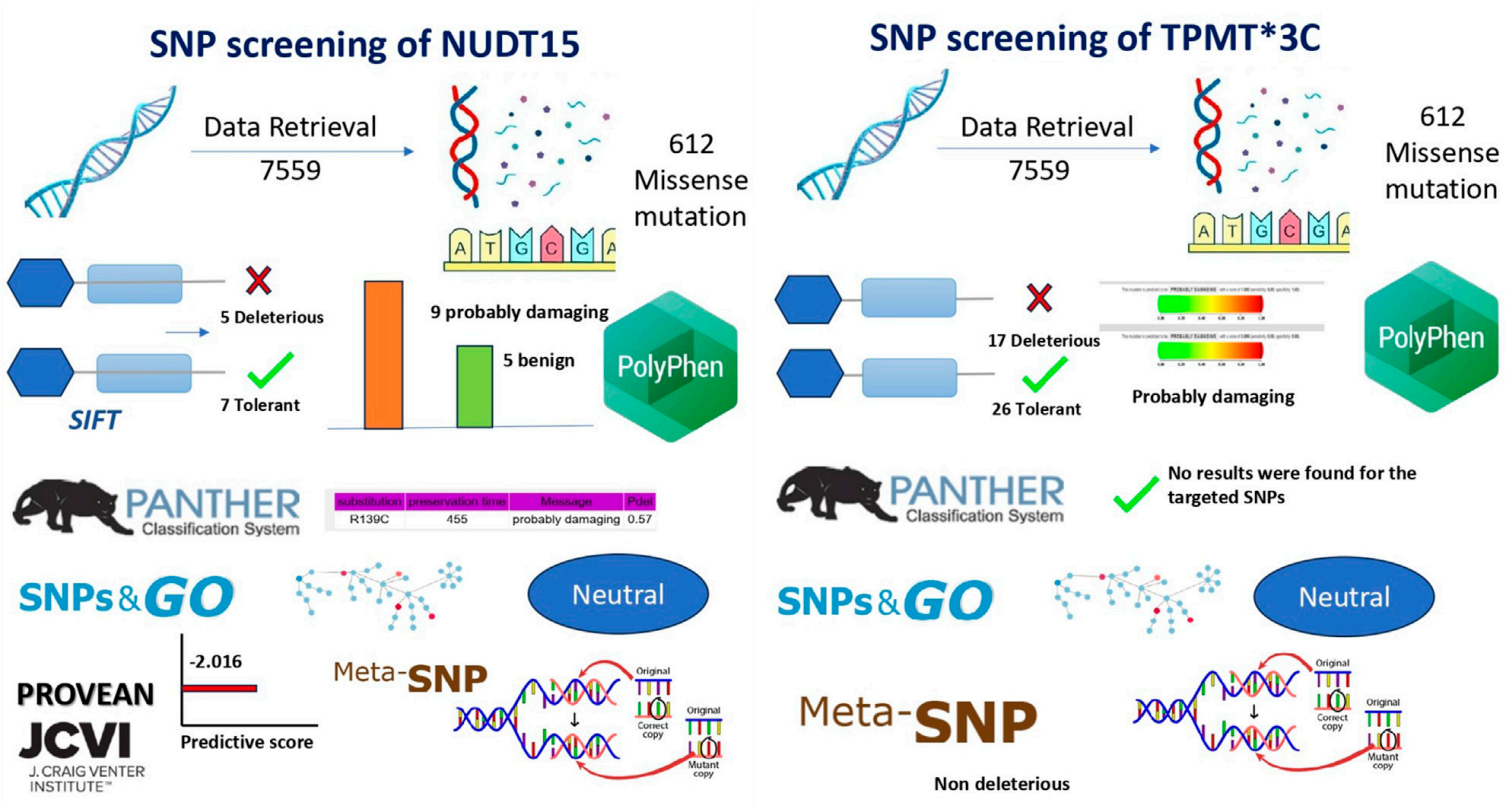
In silico analysis pipeline and predicted functional impact of NUDT15 and TPMT variants. Determination of Optimal Annealing Temperature via Gradient. Gradient PCR was performed to determine the optimum annealing temperature for the primers used in the study. Following amplification, the PCR products were analysed by agarose gel electrophoresis. Clear and distinct bands were observed at 55.9 °C, indicating efficient primer annealing and amplification at this temperature. Based on these results, 56 °C was selected as the optimum annealing temperature.

A total of 7,569 single nucleotide polymorphisms (SNPs) for the *NUDT15* gene and 11,830 for the *TPMT* gene were initially retrieved from the NCBI dbSNP database. After removal of duplicate entries (63 for *NUDT15* and 16 for *TPMT*), 7,506 unique SNPs in *NUDT15* and 11,814 in *TPMT* were retained for downstream analysis. Among these, 212 *NUDT15* and 294 *TPMT* variants were identified as missense SNPs. From this dataset, a focused subset of 12 *NUDT15* and 45 *TPMT* missense variants located within coding regions and having potential clinical relevance were systematically evaluated using multiple computational prediction tools (SIFT, PolyPhen-2, PROVEAN, SNPs&GO, and Meta-SNP) (Table 1; Figure 3). This comprehensive screening revealed that *NUDT15 c.415C>T (p.Arg139Cys)* and *TPMT*3C (c.719A>G; p.Tyr240Cys)* exhibited the most consistently deleterious predictions across all five platforms—SIFT: deleterious (0.00); PolyPhen-2: probably damaging (1.00); PROVEAN: deleterious (−6.15 and −5.25); SNPs&GO and Meta-SNP: disease-associated. Importantly, these variants also represent the most prevalent and clinically significant alleles reported among Indian pediatric ALL cohorts. The convergence of epidemiological relevance and computational pathogenicity supported their prioritization for ARMS-PCR assay development, ensuring that the diagnostic assay targets variants with both strong functional impact and clinical importance.

**TABLE 1:** List of deleterious nsSNPs in NUDT15 and TPMT genes, as identified by *in silico* tools.

S.No	Gene	Residue change	SIFT prediction (score)	PolyPhen-2 (score)	PROVEAN (score)	SNPs&GO	Meta-SNP
1	NUDT15	Val18Ile	Tolerated (0.30)	Benign (0.002)	Neutral (−1.65)	Neutral	Neutral
2	NUDT15	Arg34Thr	Deleterious (0.02)	Probably damaging (0.990)	Deleterious (−3.89)	Disease	Disease
3	NUDT15	Arg34Gln	Deleterious (0.00)	Probably damaging (0.998)	Deleterious (−4.12)	Disease	Disease
4	NUDT15	Gly47Arg	Deleterious (0.00)	Probably damaging (0.991)	Deleterious (−4.56)	Disease	Disease
5	NUDT15	Arg139Cys	Deleterious (0.00)	Probably damaging (1.000)	Deleterious (−6.15)	Disease	Disease
6	NUDT15	Arg139His	Deleterious (0.00)	Probably damaging (1.000)	Deleterious (−5.94)	Disease	Disease
7	NUDT15	Val18Leu	Tolerated (0.27)	Benign (0.016)	Neutral (−1.92)	Neutral	Neutral
8	NUDT15	Val93Ile	Tolerated (0.39)	Benign (0.003)	Neutral (−1.25)	Neutral	Neutral
9	NUDT15	Ile95Val	Tolerated (0.44)	Benign (0.012)	Neutral (−0.89)	Neutral	Neutral
10	NUDT15	Val75Glu	Tolerated (0.06)	Possibly damaging (0.658)	Neutral (−2.30)	Neutral	Neutral
11	NUDT15	Leu84Pro	Tolerated (0.21)	Possibly damaging (0.684)	Neutral (−1.96)	Neutral	Neutral
12	NUDT15	Lys107Glu	Tolerated (0.23)	Benign (0.027)	Neutral (−1.50)	Neutral	Neutral
13	NUDT15	Ala119Ser	Tolerated (0.15)	Benign (0.029)	Neutral (−1.73)	Neutral	Neutral
14	NUDT15	Arg167Gln	Tolerated (0.07)	Possibly damaging (0.704)	Deleterious (−2.67)	Disease	Disease
15	TPMT	Tyr240Cys (TPMT3C)	Deleterious (0.00)	Probably damaging (1.000)	Deleterious (−5.25)	Disease	Disease

### 3.2 Functional impact prediction using SIFT

**FIGURE 4:**
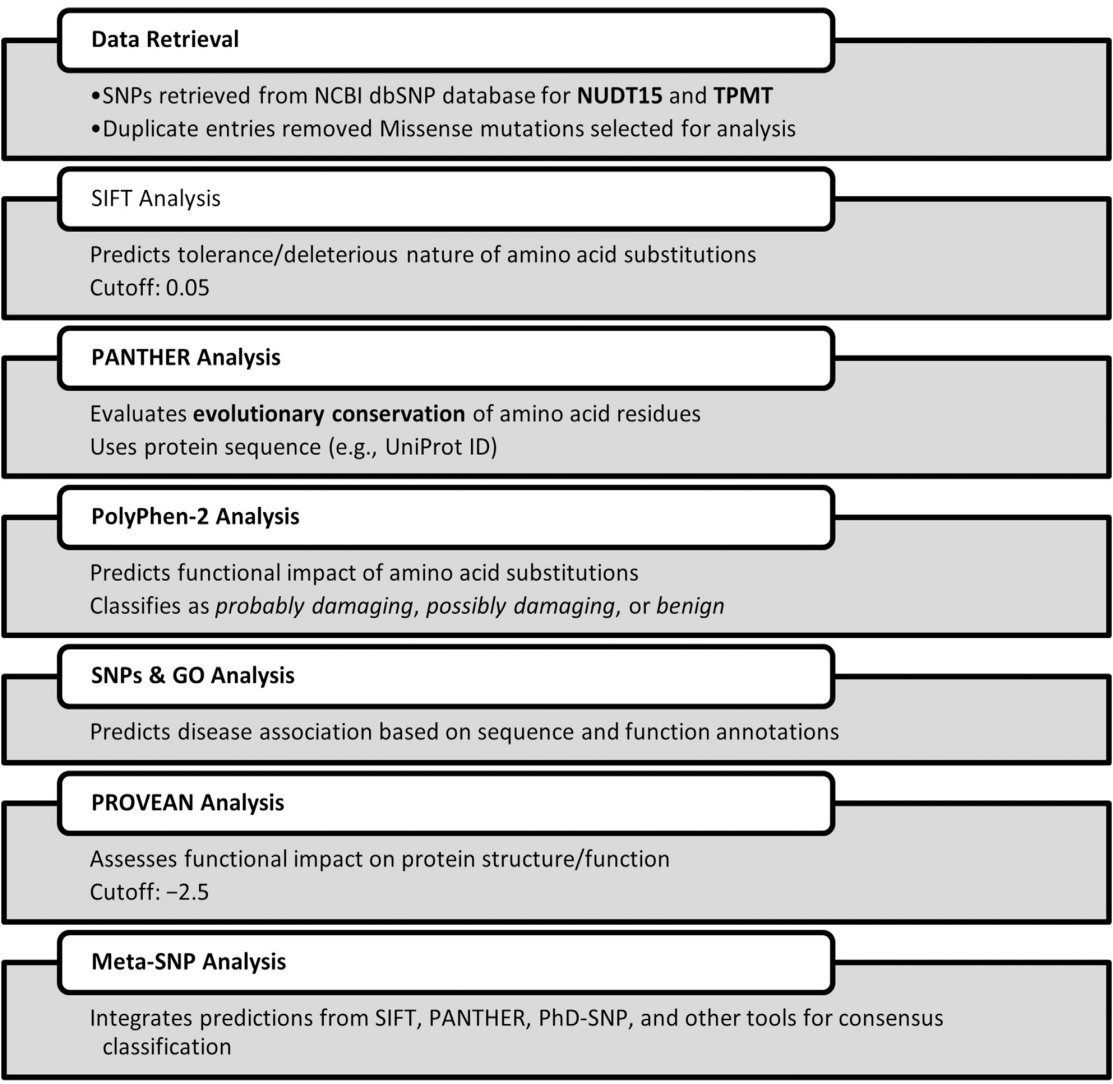
Bioinformatics workflow and pathogenicity prediction of NUDT15 and TPMT SNPs.

SIFT predicts whether an amino acid substitution is damaging based on evolutionary conservation and sequence homology. It helps identify functionally significant mutations, supporting accurate variant prioritization for personalized therapy. Functional impact analysis using SIFT classified five of the selected *NUDT15* SNPs as “deleterious” with scores below the 0.05 threshold, indicating a high likelihood of affecting protein function. The remaining seven SNPs were predicted as “tolerated.” In the case of *TPMT*, 17 SNPs were predicted as deleterious, 26 as tolerated, and one SNP could not be categorized by SIFT. These results pointed to a greater proportion of potentially damaging SNPs in *TPMT* compared to *NUDT15* (Table 1; Figure 4).

### 3.3 Evolutionary conservation analysis using PANTHER

PANTHER evaluates evolutionary conservation to identify mutations likely to disrupt protein function. It helps prioritize pathogenic variants and improve predictive accuracy for clinical relevance. Evolutionary conservation analysis using PANTHER was successfully carried out for *NUDT15*, based on the reference protein sequence (sp|Q9NV35|NUD15_HUMAN). However, the tool did not return prediction results for the selected *TPMT* variants, possibly due to limitations in its sequence alignment or conservation scoring database. Nonetheless, the available *NUDT15* data helped in prioritizing SNPs based on their conservation across species, further supporting their potential functional significance (Table 1; Figure 4).

### 3.4 Structural and functional prediction using PolyPhen-2

PolyPhen-2 was used to evaluate whether amino acid substitutions alter protein structure or function, helping to identify potentially damaging variants. This tool provided structural context to the predicted impact of NUDT15 and TPMT SNPs, supporting their potential role in thiopurine metabolism and toxicity. Among 13 successfully predicted *NUDT15* SNPs, 9 were categorized as “probably damaging,” while 4 were benign. For *TPMT*, 6 SNPs were processed, of which 3 were predicted as “probably damaging,” 2 as benign, and 1 showed no significant effect. These results aligned well with SIFT predictions and provided structural context to the potential impact of these variants (Table 1; Figure 4).

### 3.5 Disease association using SNPs&GO and Meta-SNP

To assess disease association and potential pathogenicity, both SNPs&GO and Meta-SNP tools were employed. These tools incorporate evolutionary conservation, functional domains, and machine learning algorithms. Several variants in both *NUDT15* and *TPMT* were consistently predicted to be disease-associated by both platforms, particularly those previously marked as damaging by SIFT and PolyPhen-2, strengthening the likelihood of their clinical relevance (Table 1; Figure 4).

### 3.6 Protein function disruption using PROVEAN

PROVEAN assessed whether amino acid substitutions disrupt protein function, offering an additional layer of validation for deleterious variants. Identifying functionally disruptive SNPs strengthened evidence for their potential to influence drug metabolism and adverse effects in pediatric ALL patients. Further validation was obtained using the PROVEAN tool, which predicted the effect of each amino acid substitution on protein function. Using a cutoff score of ≤ −2.5, PROVEAN categorized multiple SNPs in both *NUDT15* and *TPMT* as deleterious. These findings provided additional support for the functional relevance of key variants in both genes, suggesting that these SNPs may impact thiopurine metabolism and toxicity risk in pediatric ALL patients (Table 1; Figure 4).

The *in silico* study thus served a dual purpose following confirming the functional pathogenicity of variants already known from epidemiological studies to be prevalent in Indian ALL patients and systemically excluding other potentially damaging variants that, despite computational predictions, lacked sufficient prevalence or clinical evidence in the target population. This integrated approach combining population-specific epidemiology with computational functional predictions ensured that the developed ARMS-PCR assay addresses the most clinically actionable variants for Indian Paediatric ALL pharmacogenomics (Figure 2).

#### 3.6.1 Genotyping of NUDT15 c.415C>T and TPMT*3C variants by ARMS-PCR

**FIGURE 5:**
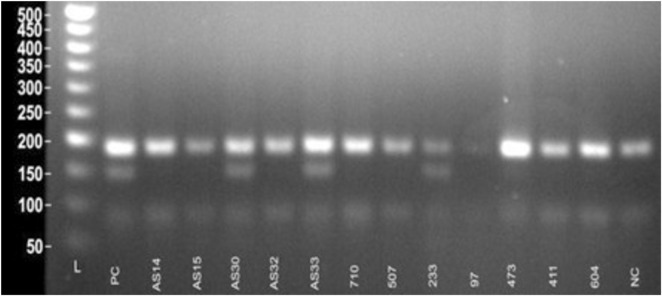
Visualization of ARMS-PCR for *NUDT15* c.415C>T variant.

Genotyping of the NUDT15 c.415C>T variant was performed using tetra-primer ARMS-PCR. Heterozygous individuals (C/T) yielded three distinct product bands of 191 bp (common amplicon), 152 bp (mutant T-allele specific), and 90 bp (wildtype C-allele specific). Wildtype homozygotes (C/C) produced bands of 191 bp and 90 bp. In the present study, among 60 pediatric ALL patients analyzed, 50 patients (83.3%) were homozygous wildtype (C/C), and 10 patients (16.7%) were heterozygous (C/T). No homozygous mutant (T/T) genotype was detected (Figure 5).

**FIGURE 6:**
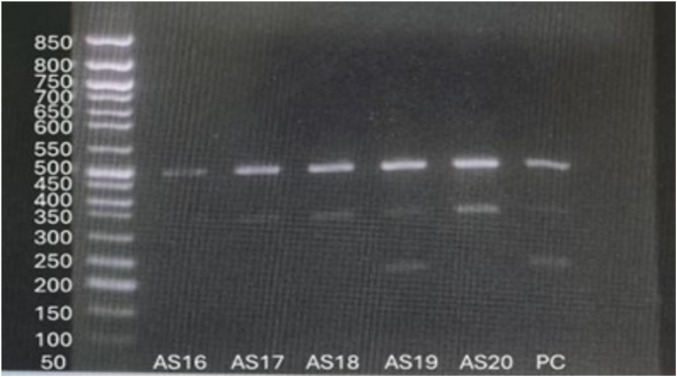
Visualization of ARMS-PCR for TPMT*3C variant.

For the TPMT*3C variant, ARMS-PCR was designed to amplify three possible genotype-specific bands: a 494 bp common amplicon, a 340 bp wildtype A-allele specific product, and a 207 bp mutant G-allele specific product. Homozygous wildtype individuals (A/A) showed 494 bp and 340 bp bands, while heterozygous individuals (A/G) exhibited all three bands (494 bp, 340 bp, and 207 bp). In this study, 58 patients (96.7%) were homozygous wildtype (A/A), and 2 patients (3.3%) were heterozygous (A/G). No homozygous mutant genotype (G/G) was observed (Figure 6).

#### 3.6.2 Comparison of ARMS-PCR with conventional sanger sequencing

**FIGURE 7:**
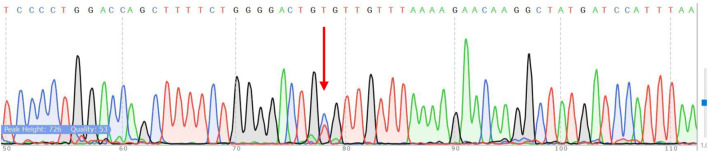
Verification of NUDT15 by Sanger sequencing. The red arrow shows the position of the point mutation.

**FIGURE 8:**
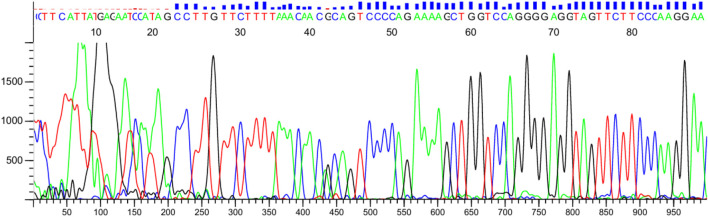
Verification of TPMT*3C by Sanger sequencing. The red arrow shows the position of the point mutation.

Sanger sequencing confirmed the ARMS-PCR genotyping results for both NUDT15 c.415C>T and TPMT*3C (c.719A>G) variants. Among the 60 pediatric ALL patients tested, 10 (16.7%) carried the NUDT15 variant allele, including 9 heterozygotes (C/T) and 1 homozygous mutant (T/T). Of these, 6 patients were alive at the time of analysis, while 4 patients had expired, reflecting the potential association of NUDT15 variants with adverse treatment outcomes (Figures 7, 8). Cascade screening of family members further identified 4 relatives who also harbored the NUDT15 variant, underscoring its heritable nature (Yang et al., 2015).

For the TPMT*3C locus, 2 patients (3.3%) were found to be heterozygous (A/G), while the remaining 58 patients were homozygous wildtype (A/A). One relative of a positive patient was also identified as a carrier of the TPMT variant. Importantly, no patient harbored both NUDT15 and TPMT variants concurrently.

The optimized ARMS-PCR assay showed high concordance (98.3%) with Sanger sequencing, confirming its reliability and diagnostic performance. A single discordant case (T/T genotype identified by sequencing but C/T by ARMS-PCR) may reflect allele dropout, emphasizing the need for sequencing confirmation in rare ambiguous cases.

##### 3.6.2.1 Diagnostic accuracy analysis

The diagnostic performance of the ARMS-PCR assay was evaluated against Sanger sequencing as the reference (gold) standard.

True positives (TP) = 10, True negatives (TN) = 50, False positives (FP) = 0, False negatives (FN) = 1 (NUDT15 T/T case undetected by ARMS-PCR).

Sensitivity = TP/(TP + FN) = 10/(10 + 1) = 90.9%

Specificity = TN/(TN + FP) = 50/(50 + 0) = 100%

Diagnostic accuracy = (TP + TN)/Total = 60/61 = 98.4%

These findings indicate high specificity and acceptable sensitivity, confirming the assay’s potential for clinical screening use.

To evaluate the diagnostic performance of the ARMS-PCR assay, sensitivity, specificity, positive predictive value (PPV), negative predictive value (NPV), and overall accuracy were calculated using Sanger sequencing as the gold standard reference method. Diagnostic parameters were calculated using standard formulas with 95% confidence intervals.

#### 3.6.3 Comparison of blast cell percentage at baseline and follow-up

**FIGURE 9:**
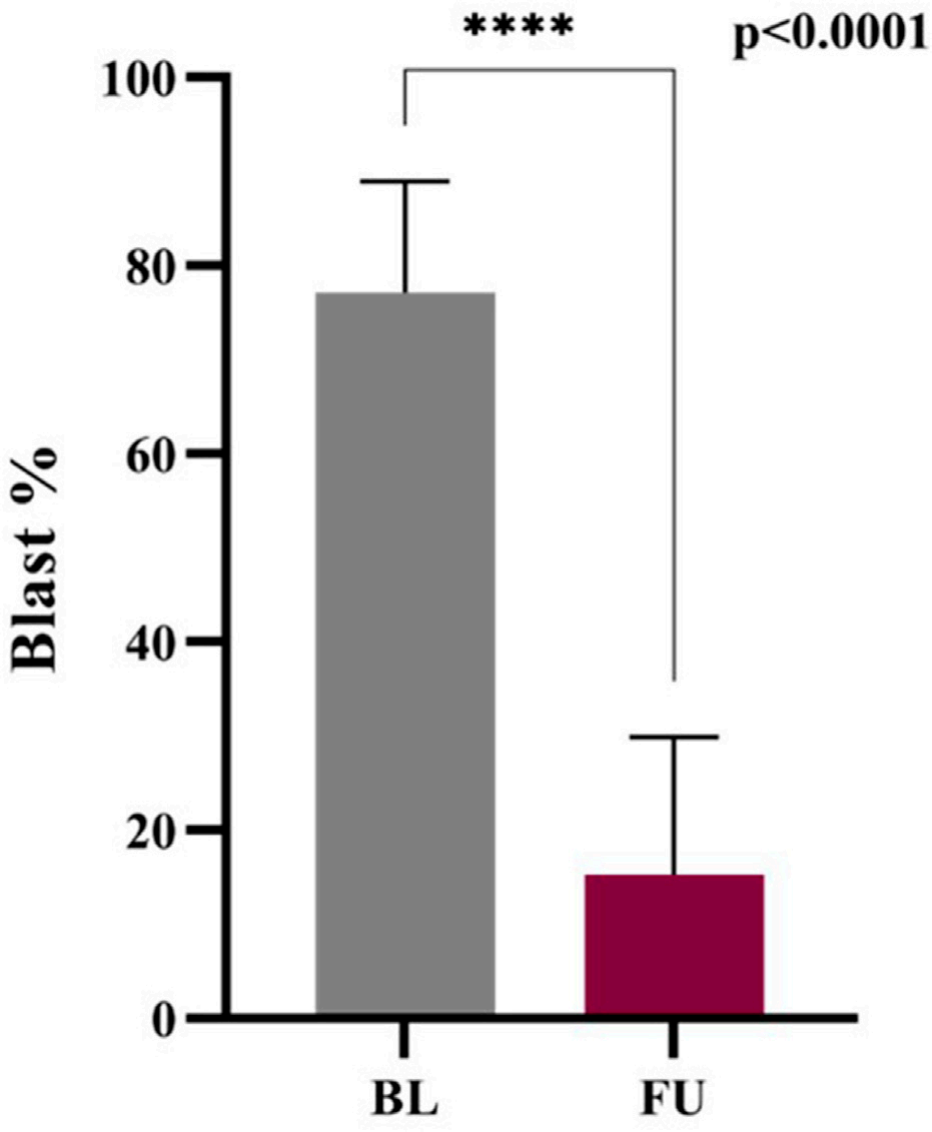
Blast percentage: Baseline vs. Follow-up.

This plot explained the change in blast cell percentage in baseline and follow-up of ALL patients. There is a huge decrease from 75% to 15% (95% CI −67.02 to −56.68) in the blast cell percentage of baseline and follow-up patients. *P*-value (0.0001, MannWhitney test) is highly significant and shows the treatment response and remission status in the follow-up patients (Figure 9). Early blast clearance is a recognized prognostic marker. Studies show that quickly reducing blasts is linked to better relapse-free and overall survival (Jena et al., 2021; Zgheib et al., 2020) (Park et al., 2018; Buchmann et al., 2022). Monitoring blast cell percentages offers a dependable way to measure treatment effectiveness and helps inform clinical decisions in ALL (Supplementary Table S1).

#### 3.6.4 Analysis of 6-mp dose intensity in *nudt15* wildtype and *nudt15* mutant

**FIGURE 10:**
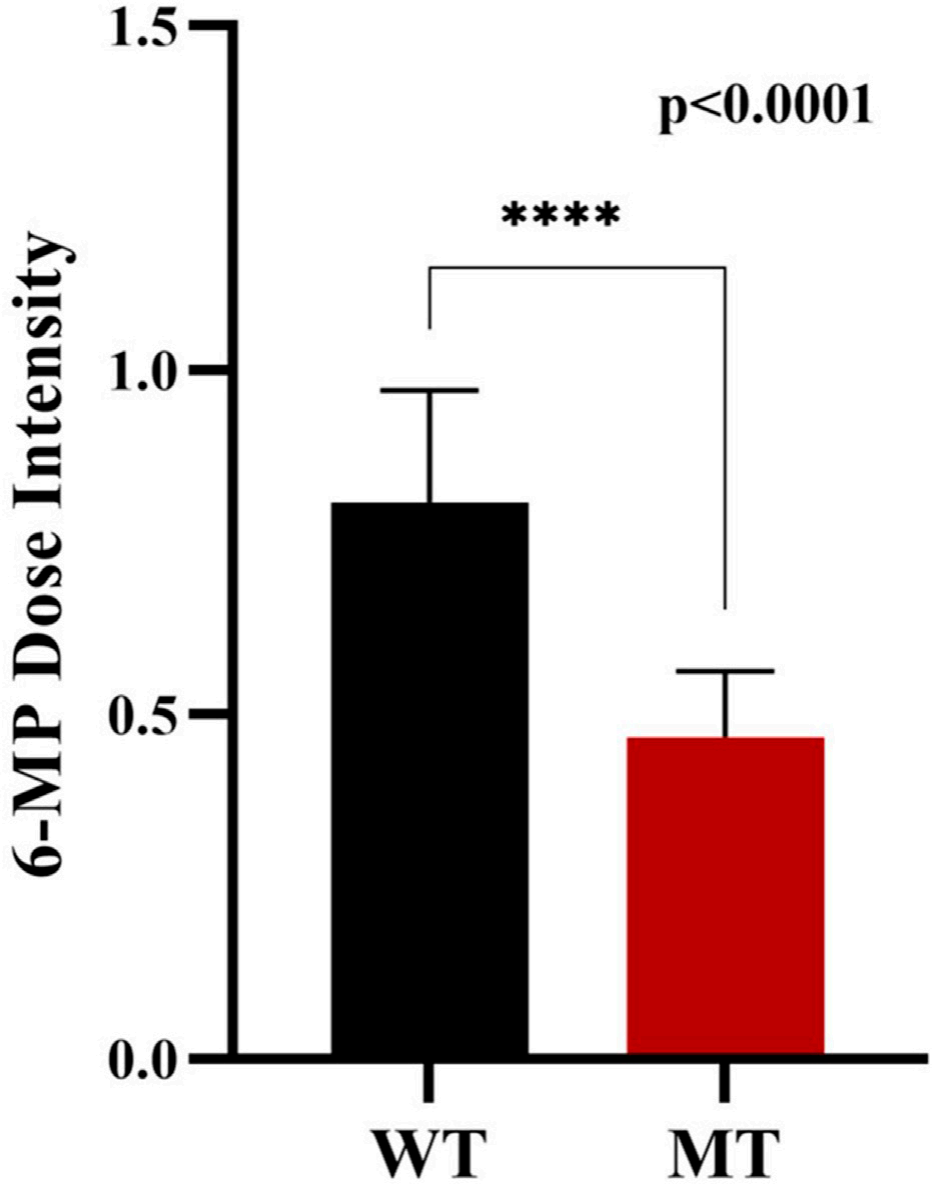
6-Mp dose intensity: Wild-type and *NUDT15* mutant-type.

The Wildtype patients show higher mean dose intensity of 6-MP whereas mutant type shows comparatively lower mean dose intensity of 6-MP. Patients with *NUDT15* mutation (MT) receive a significantly lower dose of intensity due thiopurine intolerance associated with *NUDT15*. The planned dose of 6-MP given to ALL patients is 60 mg/m^2^. The median dose for wildtype patients is 0.79 (47.4 mg/m^2^) and for mutant type patient is 0.5 (30 mg/m^2^). The marked statistical difference (*p* < 0.0001) confirms that his dosage adjustment is consistent and not random (Figure 10).

## 4 Discussion

In this study, we successfully developed and validated a cost-effective, reproducible tetra-primer ARMS-PCR assay for detecting NUDT15 c.415C>T (rs116855232) and TPMT 3C (c.719A>G; rs1142345) variants in Indian paediatric ALL patients. The assay demonstrated 98.3% concordance with Sanger sequencing, confirming its accuracy and reliability. Furthermore, the assay required minimal DNA input, with a limit of detection as low as 10 ng, underscoring its sensitivity and suitability for routine clinical use in settings where DNA availability may be limited (Ho et al., 2017; Lone et al., 2023).

An important component of our study was the use of comprehensive *in silico* analyses to prioritize *NUDT15* and *TPMT* variants before wet lab validation. We employed multiple computational tools including SIFT, PolyPhen-2, PROVEAN, Meta-SNP, and SNPs and GO to assess conservation, structural impacts, and functional consequences of missense substitutions. Across these tools, *NUDT15 c.415C>T* (p.Arg139Cys) and *TPMT 3C* (p.Tyr240Cys) consistently scored as deleterious, predicted to destabilize protein folding or impair enzymatic function. These predictions are supported by recent bioinformatics studies such as Saxena et al. (2022), Jena et al. (2021), which identified known and novel TPMT missense SNPs predicted deleterious by *in silico* pipelines and molecular dynamics simulations; (Cook et al., 2023; Zgheib et al., 2020), who compared genotypes and phenotypes for *TPMT* and *NUDT15* in a large pediatric cohort and found strong concordance between predicted functional impact and observed toxicity; and Zhou et al. (2020), Liang et al. (2016), who demonstrated high accuracy of algorithms in predicting *in vivo* consequences of TPMT variants.

These bioinformatics platforms support detailed computational assessments of missense variants. They predict potential effects on protein structure and function. By combining evaluations of evolutionary conservation, structural integrity, and functional consequences, these platforms help prioritize variants that are most likely to affect drug metabolism and treatment effectiveness. This method is crucial for identifying candidate variants for testing. It also improves resource allocation and speeds up the research process in pharmacogenomics. By coupling these computational predictions with our ARMS-PCR assay selection, we ensured that the variants tested are both biologically relevant and clinically actionable, particularly in a resource‐limited setting.

The primary contribution of this work lies in the validation of ARMS-PCR as a diagnostic tool. Unlike sequencing methods, which require costly equipment and skilled manpower, ARMS-PCR provides a rapid, low-cost alternative that can be easily implemented in regional laboratories. This makes it particularly relevant in the Indian healthcare setting, where widespread pharmacogenetic testing remains limited due to resource constraints (Tanaka and Saito, 2021; Mao et al., 2021).

In our cohort, the prevalence of the NUDT15 variant allele (∼15%) was substantially higher than that of TPMT 3C (∼1.7%). This aligns with previous Indian studies, which reported NUDT15 allele frequencies of 10%–16% and TPMT 3C in the range of 2%–3.5% (Valerie et al., 2016; Aşlar Öner, 2024; Weinshilboum and Sladek, 1980). Similar trends have been observed in East Asian populations, where NUDT15 but not TPMT strongly predicted thiopurine intolerance (Valerie et al., 2016; Iqbal et al., 2018). These findings reaffirm the greater clinical impact of NUDT15 polymorphisms in South and East Asian cohorts compared to Western populations, where TPMT variants are the predominant determinant of thiopurine toxicity (Umadevi et al., 2025).

Similar approaches utilizing ARMS-PCR for pharmacogenetic screening have been reported previously. Ho et al. developed and validated tetra-primer ARMS-PCR assays for the detection of NUDT15 c.415C>T and TPMT*3C (c.719A>G) variants in East Asian populations, demonstrating complete concordance with conventional Sanger sequencing and highlighting its cost-effectiveness for clinical application (Ho et al., 2017). In the Indian context, Shah et al. employed ARMS-PCR and restriction fragment length polymorphism (RFLP) assays for preemptive NUDT15 genotyping, reporting high sensitivity and specificity in identifying patients at risk of thiopurine-induced leukopenia (Lone et al., 2023) (Other groups have similarly adapted allele-specific PCR strategies, including high-resolution melt analysis, RFLP, and TaqMan-based assays, to achieve rapid, accurate, and affordable genotyping of TPMT and NUDT15 variants, further underscoring the clinical utility of low-cost molecular platforms in resource-limited settings (Park et al., 2018; Saxena et al., 2022).

Mechanistically, NUDT15 encodes an enzyme that hydrolyzes thioguanosine triphosphate (TGTP) into its monophosphate derivative, preventing DNA incorporation. The c.415C>T variant destabilizes the protein, leading to TGTP accumulation and early-onset myelosuppression (Umadevi et al., 2025). Conversely, TPMT catalyzes S-methylation of thiopurines, and the 3C variant reduces enzyme activity but is rare in Asians (Pratt et al., 2022). Clinical evidence from Indian and Asian cohorts consistently demonstrates that NUDT15 carriers are at significantly higher risk of severe leukopenia, hair loss, and intolerance to standard 6-mercaptopurine doses, often necessitating dose reductions to 10%–30% of the conventional starting dose (Khera et al., 2019; Valerie et al., 2016; Lee et al., 2021).

Beyond pharmacogenetic screening, improvements in parallel diagnostic tools also show the clinical value of combining different technologies. For example, using flow cytometry for immunophenotypic prognostication of acute promyelocytic leukemia has demonstrated its benefits (Siraj et al., 2021). This is similar to how initial TPMT/NUDT15 genotyping guides therapeutic safety in ALL.

The Clinical Pharmacogenetics Implementation Consortium (CPIC) guidelines recommend pre-emptive genotyping of both NUDT15 and TPMT to guide thiopurine dosing (Mao et al., 2021). However, in India, pharmacogenetic testing is not routinely performed before therapy initiation. Our assay provides a feasible solution to bridge this gap, enabling clinicians to identify high-risk patients upfront and optimize thiopurine therapy accordingly.

Compared to sequencing-based platforms, our ARMS-PCR assay offers several advantages. It is highly cost-effective, reducing expenses by more than 70% compared to sequencing (Tanaka and Saito, 2021; Lone et al., 2023), and provides a rapid turnaround time, with genotyping results available within a single working day. The method requires only basic PCR and electrophoresis equipment, making it easily adaptable to peripheral laboratories with limited infrastructure. Importantly, the assay demonstrated high diagnostic accuracy, with complete concordance to sequencing in this study. Together, these strengths make ARMS-PCR particularly attractive for scaling pharmacogenetic testing in low-resource settings, which is essential for the equitable implementation of precision oncology in India.

The overall cost of ARMS-PCR is considerably lower than that of Sanger sequencing. ARMS-PCR requires only specific primers, a standard PCR mastermix, and a conventional thermal cycler, whereas Sanger sequencing involves additional steps such as post-PCR purification, specialized reagents, and access to a capillary sequencer, which significantly increases both consumable and equipment costs. Thus, ARMS-PCR offers a markedly more economical alternative without compromising diagnostic utility.

Future research should investigate the integration of molecular assays with emerging technologies, including nanoplatform-mediated imaging, to advance oncological diagnostics. Although nanoplatforms offer advanced and multifunctional diagnostic capabilities, cost-effective molecular assays are crucial for personalizing therapies in resource-constrained settings. Additionally, drug repurposing strategies, such as the observed effect of letrozole on seizure modulation without increasing neurotoxicity, highlight the importance of pharmacogenetic knowledge in improving therapeutic safety across diverse medical disciplines (Wang et al., 2024; Dave et al., 2024).

A key strength of this study is the integration of bioinformatics analysis with laboratory validation, ensuring that clinically relevant variants were prioritized for testing. Additionally, family-based cascade screening confirmed the heritability of NUDT15 variants, supporting their clinical significance. While ARMS-PCR for NUDT15 and TPMT variants has been reported in other populations, this is the first study to integrate *in silico* variant prioritization with clinical validation on an Indian pediatric ALL cohort, establishing population-specific diagnostic utility. Moreover, we optimized reaction conditions for low DNA input (10 ng), demonstrating assay reproducibility and scalability in real-world hospital setting.

## 5 Conclusion

In conclusion, this study validates ARMS-PCR as a robust and cost-effective assay for *NUDT15* and *TPMT* genotyping in Indian paediatric ALL patients. Given the higher prevalence and stronger clinical impact of *NUDT15* compared to *TPMT* in South Asians, integrating this assay into treatment protocols could enable genotype-guided thiopurine dosing, improve patient safety, and reduce treatment interruptions. Our findings demonstrate the feasibility of adopting pharmacogenomic screening in resource-limited healthcare systems, marking an important step toward equitable precision oncology in India.

Nonetheless, the study has certain limitations. Only two variants were examined (*NUDT15 c.415C>T* and *TPMT 3C*), although additional alleles such as *TPMT 3A*, *3B*, and *NUDT15 p.Arg34Thr* may also influence thiopurine toxicity. The single-centre design with a modest sample size may not capture the full spectrum of regional genetic diversity, and prospective genotype–toxicity correlations were not systematically assessed. Future work should expand variant coverage, validate findings across larger multicentric cohorts, and perform longitudinal monitoring to refine genotype-guided dosing strategies. Cost-effectiveness analyses in real-world practice will be essential to support the routine integration of ARMS-PCR into paediatric oncology care in India.

## Data Availability

The original contributions presented in the study are included in the article/Supplementary Material, further inquiries can be directed to the corresponding authors.
