# Supplementary material for: Validation of diagnostic screening test for pharmacogenomic targets for thiopurine drugs in indian pediatric acute lymphoblastic leukemia patients
Source: Front Pharmacol. 2025 Dec 8;16:1714797. doi: 10.3389/fphar.2025.1714797 (PMC12722899; doi:10.3389/fphar.2025.1714797)
Supplement: Supplementary file 1 [file Table2.docx]

Supplementary Material

# Supplementary Tables

**Supplementary Table 1:** Primer Datasheet for TPMT

| ***TPMT*** | | | |
| --- | --- | --- | --- |
| **Type** | **PRIMERS** | **Tm ℃** | **Expected**  **Product**  **Size (bp)** |
| ***TPMT* c.719A>G (*TPMT*3C*)** | | | |
| T-OF | 5′-CACCCAGCCAATTTTGAGTA-3′ | 49.7 | 494 |
| T-OR | 5′-CAGGTAACACATGCTGATTGG-3′ | 52.4 | 494 |
| T-MT | 5′-ATGTCTCATTTACTTTTCTGTAAGTACAC-3′ | 54.4 | 207 |
| T-WT | 5′-TTGACTGTCTTTTTGAAAAGTTCTA-3′ | 49.5 | 340 |

**Supplementary Table 2:** Primer datasheet for NUDT15

| **Primer** | **Sequence** | **Tm**  **(°C)** | **Expected**  **Product**  **Size (bp)** | **Final**  **Concentration**  **(μM)** |
| --- | --- | --- | --- | --- |
| ***NUDT15* c.415C>T genotyping** | | | | |
| N-OF | 5′-CCCAAATAAACACCCTTTGTTTTCTGT-3′ | 55.2 | 191 | 0.18 |
| N-OR | 5′-CCTTTGTATCCCACCAGATGGTTC-3′ | 57.4 | 191 | 0.18 |
| N-WT | 5′-GGACCAGCTTTTCTGGGGACTAC-3′ | 58.8 | 90 | 0.82 |
| N-MT | 5′-GGATCATAGCCTTGTTCTTTTAAACAATA-3′ | 54.4 | 152 | 0.82 |
